# Supplementary material for: Investigating the Feasibility of Predicting KRAS Status, Tumor Staging, and Extramural Venous Invasion in Colorectal Cancer Using Inter-Platform Magnetic Resonance Imaging Radiomic Features
Source: Diagnostics (Basel). 2023 Nov 27;13(23):3541. doi: 10.3390/diagnostics13233541 (PMC10706390; doi:10.3390/diagnostics13233541)
Supplement: Supplementary file 1 [file diagnostics-13-03541-s001.zip › diagnostics-2630260-supplementary.pdf]

Supplementary Table S1. Clinical and Treatment Characteristics of Colorectal Cancer Patients. This table provides detailed information on key clinical parameters and treatment regimens for colorectal cancer patients. Patient data includes unique codes, gender, weight, age, KRAS mutation status, TNM staging, effects on mesorectal fascia (MRF), extramural vascular invasion (EMVI), and type of treatments received.

#	Patient Code Number	SEX	weight	age	KRAS (N/P)	Tumour staging (TNM staging system)	T stage	N stage	Metastasis	Effects of mesorectal fascia (MRF) (N/P)	Extramural vascular invasion (EMVI) (N/P)	Type of treatments
1	10172719	F	61	42	N	T4b, N2, M0/ yT4aN0Mx	T4b	N2	M0	yes	Positive	3 cycles of NAD XELOX
2	60661	M	80	54	N	T3N2M0/ after treatment T1 N1	T3	N2	MO	NO	Negative	Neoadjuvant chemoradiotherapy
3	340433	M	110	62	N	T3N2	T3	N2	-	NO	Negative	Neoadjuvant chemoradiotherapy
4	449272	F	78	58	N	t4n2	T4	N2	-	Yes	Positive	CHEMO (XELOX) X 2
5	2322853	M	57	45	N	T4b, N2, M0/ NO FU	T4b	N2	MO	Yes	Positive	No data
6	2539669	F	85	50	N	T3 N2	T3	N2		Yes	Positive	Neoadjuvant chemoradiotherapy
7	10010705	M	81	55	N	T4AN2/ T3N0Mx after treatment	T4A	N2	M0	Yes	Positive	Neoadjuvant chemoradiotherapy
8	10087230	M	55	67	N	T2N2/ NO FU	T2	N2	M0	No	Negative	CHEMO (XELOX) X 2
9	10089371	M	100	80	N	T4AN2/ NO FU	T4A	N2	M0	Yes	Negative	CHEMO (XELOX) X 3
10	10096833	M	75	57	N	T2N2 / AFTER TREAT T1-2 N2	T2	N2	M0	No	Negative	Neoadjuvant chemoradiotherapy
11	10103691	M	100	44	N	T3N2 / AFTER TREATMENT T3N2	T3	N2	M0	Yes	Positive	Neoadjuvant chemoradiotherapy
12	10134636	M	90	57	N	T2N1/ AFTER T2N1	T2	N1	M0	No	Negative	Neoadjuvant chemoradiotherapy
13	10135968	M	80	46	N	T4N2M1	T4	N2	M1	Yes	Positive	Neoadjuvant chemoradiotherapy
14	10138041	M	85	69	N	T4 N2 M0/ NO FU	T4	N2	M0	Yes	-	-
15	10139959	F	47	50	N	T3 N0	T3	N0	M0	Yes	Negative	cycles of NAD XELOX
16	10140593	M	70	73	N	T4B N2/ NO FU	T4B	N2	M0	Yes	Positive	No data
17	10143049	F	100	52	N	T3N2M0	T3	N2	M0	Yes	Negative	CHEMO (XELOX) X 2
18	10179989	M	50	50	N	T3N0	T3	N0	M0	Yes	Positive	CHEMO (XELOX) X 3
19	10184428	F	98	57	N	T3 N0 MX/ypT1 N0 Mx	T3	N0	M0	No	Negative	CHEMO (XELOX) X 4
20	10188227	M	70	49	N	T3N2	T3	N2	M0	Yes	Positive	Xeloda
21	317873	M	110	62	N	T2N2M0 / SURGERY	T2	N2	M0	Yes	Negative	-
22	534327	M	85	61	N	T4N2/ AFTER TREAT ALMOST GONE	T4	N2	M0	Yes	Positive	Neoadjuvant chemoradiotherapy
23	1135947	F	55	46	N	T3 N1 M0	T3	N1	M0	Yes	Positive	-

**24**	**1199271**	**M**	**81**	**44**	**N**	T2N2M0/ NO FU	T3	N0	M0	No	Positive	Neoadjuvant chemoradiotherapy
**25**	**2132915**	**M**	**66**	**59**	**N**	T3 N2 M1 / NO FU	T3	N2	M1	Yes	Positive	Neoadjuvant chemoradiotherapy
**26**	**10050980**	**F**	**85**	**64**	**N**	T4B N2 M1/ yT4N0/1	T4B	N2	M1	-	Positive	chemo
**27**	**10051270**	**F**	**90**	**71**	**N**	T4N2M0/ NO FU	T4	N2	M0	-	Positive	chemo
**28**	**10205635**	**M**	**70**	**54**	**N**	T3N2M0/ NO FU	T3	N2	M0	Yes	Positive	CHEMO (XELOX) X 4
**29**	**10206130**	**M**	**55**	**53**	**N**	T3/ NO FU	T3	N0	M0	-	Positive	Chemo
**30**	**10207469**	**F**	**85**	**52**	**N**	T4bN2M1/ No FU	T4B	N2	M1	-	Positive	Neoadjuvant chemoradiotherapy
**31**	**10214476**	**F**	**70**	**44**	**N**	T3N2M0	T3	N2	MO	Yes	Positive	Neoadjuvant chemoradiotherapy
**32**	**10216119**	**F**	**80**	**61**	**N**	T3N2/ NO FU	T2	N2	M0	Yes	Positive	chemo
**33**	**10233788**	**M**	**100**	**57**	**N**	T4N2M1/ NO FU	T4	N2	M1	Yes	Positive	-
**34**	**140777**	**F**	**79**	**59**	**P**	T2N2M1	T2	N2	M1	Yes	Negative	Neoadjuvant chemoradiotherapy
**35**	**340243**	**M**	**87**	**47**	**P**	T4AN2M0 / NO FU	T4A	N2	M0	Yes	Positive	Neoadjuvant chemoradiotherapy
**36**	**368356**	**F**	**80**	**59**	**P**	T4N2M0/ NO FU	T4	N2	M0	Yes	Positive	Neoadjuvant chemoradiotherapy
**37**	**502757**	**M**	**90**	**69**	**P**	T3 N 2 M0 / yT3, N1, Mx	T3	N2	M0	No	Positive	Neoadjuvant chemoradiotherapy
**38**	**544515**	**M**	**95**	**62**	**P**	T4AN2M0	T4A	N2	M0	No	Negative	chemo
**39**	**2236146**	**M**	**70**	**45**	**P**	T3N1 M0 / AFTER TREAT T4 N2 M0	T3	N1	M0	Yes	Positive	Neoadjuvant chemoradiotherapy
**40**	**2443955**	**M**	**75**	**60**	**P**	T4N2M0	T4	N2	M0	Yes	Positive	Neoadjuvant chemoradiotherapy
**41**	**10015390**	**M**	**52**	**96**	**P**	T3 N 2 M0	T3	N2	M0	No	Positive	chemo
**42**	**10019202**	**M**	**78**	**80**	**P**	T2-T3 N2 M0/ AFTER TREAT T2N2M0	T2-T3	N2	M0	No	Positive	-
**43**	**10026773**	**M**	**99**	**65**	**P**	T4N1M0	T4	N1	M0	Yes	Positive	Neoadjuvant chemoradiotherapy
**44**	**10076837**	**M**	**45**	**59**	**P**	T4N1M1/ NO FU	T4	N1	M0	Yes	Positive	Neoadjuvant chemoradiotherapy
**45**	**10150880**	**F**	**53**	**41**	**P**	T4N2 M0	T4	N2	M0	Yes	Positive	chemo
**46**	**10155918**	**M**	**60**	**75**	**P**	T3N1M1	T3	N1	M1	Yes	Positive	Neoadjuvant chemoradiotherapy
**47**	**432974**	**M**	**100**	**54**	**P**	T3N2/ Interval reduction on size with no change on stage	T3	N2	M0	no	Positive	Neoadjuvant chemoradiotherapy
**48**	**450518**	**M**	**74**	**65**	**P**	T2N0M0/ NO FU	T2	N0	M0	no	Positive	Neoadjuvant chemoradiotherapy
**49**	**1207020**	**F**	**55**	**69**	**P**	T3 N1 / NO FU	T3	N1	M0	no	Negative	Neoadjuvant chemoradiotherapy
**50**	**10047266**	**F**	**80**	**51**	**P**	T4 N1 M1/ POST SURGERY	T4	N1	M1	Yes	Positive	Neoadjuvant chemoradiotherapy
**51**	**10057014**	**F**	**51**	**46**	**P**	T3N1M0 / NO FU	T3	N1	M0	no	Positive	Neoadjuvant chemoradiotherapy
**52**	**10096937**	**F**	**66**	**61**	**P**	T3N2/ POST SURGERY	T3	N1	M0	Yes	Positive	chemo
**53**	**10134812**	**M**	**70**	**57**	**P**	T2 N2/ NO FU	T2	N2	M0	no	Negative	chemo

**54**	**10147507**	**F**	**80**	**59**	**P**	T4 N2 M0	T4	N2	M0	no	Negative	Neoadjuvant chemoradiotherapy
**55**	**10208084**	**F**	**75**	**62**	**P**	T3N2/ AFTER TREATMENT T3 N2	T3	N2	M0	no	Positive	Neoadjuvant chemoradiotherapy
**56**	**10214476**	**F**	**70**	**44**	**P**	T3 N2 M0	T3	N2	M0	Yes	Positive	Neoadjuvant chemoradiotherapy
**57**	**176761**	**M**	**85**	**51**	**P**	T4AN2M0 / NO FU	T4A	N2	M1	Yes	Positive	chemo
**58**	**517223**	**F**	**55**	**23**	**P**	T4B N2 M1/ NO FU	T4B	N2	M1	no	Positive	chemo
**59**	**10246553**	**M**	**62**	**32**	**P**	T3N2	T3	N2	M0	no	Negative	Neoadjuvant chemoradiotherapy
**60**	**10254537**	**F**	**35**	**13**	**P**	T3N2 M0	T3	N2	M0	Yes	Negative	Neoadjuvant chemoradiotherapy
**61**	**10256859**	**F**	**50**	**56**	**P**	T4bN2M1/ Post surgery	T4B	N2	M1	-	-	CONCOMITTENT chemo rad
